# Transcriptomic and Metabolomic Differences Between Two *Saposhnikovia divaricata* (Turcz.) Schischk Phenotypes With Single- and Double-Headed Roots

**DOI:** 10.3389/fbioe.2021.764093

**Published:** 2021-10-28

**Authors:** Tao Zhang, Yuqiu Chen, Qinghe Zhang, Peng Yu, Qiong Li, Weichen Qi, Changbao Chen

**Affiliations:** Key Laboratory of Chinese Medicine Planting and Development, Changchun University of Chinese Medicine, Changchun, China

**Keywords:** *Saposhnikovia divaricata*, transcriptome, metabolite, biotechnology, chromone

## Abstract

*Saposhnikovia divaricata* is derived from the dried roots of *Saposhnikovia divaricata* (Turcz.) Schischk and used as a Chinese herbal medicine for treating respiratory, immune, and nervous system diseases. The continuously increasing market demand for traditional Chinese medicine requires the commercial cultivation of *Saposhnikovia divaricata* using standardized methods and high yielding genotypes, such as double-headed root plants, for achieving consistent quality and a reliable supply. In this study, we aimed to identify the quantitative differences in chromone, a precursor of flavonoid biosynthesis, between plants with single- and double-headed roots using high-performance liquid chromatography and further explore the two phenotypes at the transcriptomic and metabolomic levels. Our results showed that the chromone content was significantly higher in plants with double-headed roots than in those with single-headed roots. Transcriptomic analysis revealed six significantly differentially expressed genes between the two phenotypes, including five key genes in the flavonoid biosynthesis pathway (*4-coumarate-CoA ligase*, *chalcone synthase 1*, *vinorine synthase*, *chalcone-flavonone isomerase 1*, and *flavanone 3 beta-hydroxylase*) and one key gene in the abscisic acid biosynthetic pathway (zeaxanthin epoxidase). Moreover, metabolomic analysis showed that the 126 differentially expressed metabolites were mainly enriched in the biosynthesis of secondary metabolites and phytohormones. Overall, our results suggest that plants with double-headed roots have higher medicinal value than those with single-headed roots, probably due to differences in various biosynthetic pathways. These data might help select the genotypes with superior yield and therapeutic properties.

## Introduction

*Saposhnikovia divaricata*, also known as Fangfeng, is derived from the dried roots of *Saposhnikovia divaricata* (Turcz.). Schischk and used as a Chinese herbal material owing to its antipyretic, analgesic, antioxidant, antiproliferative, and anti-inflammatory properties (Khan et al., 2011; Okuyama et al., 2019). The chemical composition of *S. divaricata* is complex and includes more than 100 compounds, such as chromone, which is a precursor of flavonoid biosynthesis, coumarin, and volatile oil. Previous studies have shown that the root has the highest medicinal value during the vegetative stage (Liu et al., 2020), which is significantly reduced in the reproductive stage (Li et al., 2017). Therefore, it is necessary to determine the medicinal substances of *S. divaricata* and elucidate the underlying formation mechanism (Liu and Zhang, 2007).

Transcriptomics is the study of gene expression at the RNA level under specific physiological conditions and helps identify the differentially expressed genes (DEGs) (Armstrong and Que, 2012; Hamilton and Buell, 2012; Ramaswami et al., 2013) and understand the molecular basis of phenotypic differences (Forrest and Carninci, 2009; Rodríguez-García et al., 2017). Transcriptomics has been used for gene mining, function prediction, and metabolic pathway analysis of various medicinal plants, including *Panax ginseng* (Hongzhe et al., 2015; Li et al., 2013; Liu et al., 2016; Zhang et al., 2021), *Panax quinquefolius* (Wu et al., 2013; Zou et al., 2015), *Scutellaria baicalensis* (Cheng et al., 2018), *Salvia miltiorrhiza* (Gao et al., 2014), and *Bupleurum chinense* (Sui et al., 2011). Transcription factors initiate and regulate gene expression by recognizing and binding to cis-acting elements in the promoter region of genes. Studies have shown that WRKY, bHLH, and bZIP transcription factors play important roles in defense responses against various abiotic stresses (Madhunita and Ralf, 2014), in plant growth, stress resistance, and signal transduction (Liu et al., 2015), and in plant development and physiological metabolic processes (Zhang et al., 2011), respectively. Therefore, genome-wide transcriptomics, together with transcription factor analysis, allows us to understand plant responses via transcriptional changes and elucidate the molecular mechanisms underlying the biosynthesis of secondary metabolites in medicinal plants.

Metabolomics is the comprehensive qualitative and quantitative characterization of all endogenous small molecule metabolites in biological systems. It has been widely applied to study metabolites in medicinal plants due to its high sensitivity, resolution, accuracy, and wide dynamic range. Phytohormones are signal molecules that are naturally present in plants in extremely low concentrations, influencing plant growth and development (Hong and Xu, 2013). Of these, brassinolide, salicylic acid, and jasmonic acid are involved in plant-pathogen interactions (Jameson et al., 2016); cytokinin (CTK) is a derivative of adenine and, together with indolyl acetic acid, induces plant cell division, inhibits leaf senescence, and defends against abiotic stress (Werner and Schmulling 2009); zeatin nucleoside, which is the main form of CTK transported in the xylem, controls the differentiation of plant flower buds; and abscisic acid (ABA) regulates plant and environmental signals (Zhang et al., 2010). Plant endogenous hormones play an important role in the process of plant growth and development, which may be the cause of the double-headed roots of *S. divaricata*.

The market demand for traditional Chinese medicine continues to grow worldwide. Thus, the commercial cultivation of *S. divaricata* using standardized methods is required for achieving consistent quality and a reliable supply. The conventional cultivation of *S. divaricata* includes plants with single-headed roots (one aerial part); however, we found that those with double-headed roots (two aerial parts) have a higher yield potential. In this study, we aimed to identify the differences in the chromone content between plants with single- and double-headed roots using high-performance liquid chromatography and further explore the two phenotypes via transcriptomic and metabolomic analyses. Our study provides an important theoretical basis for the quality research of *S. divaricata* medicinal materials.

## Materials and Methods

### Plant Material

**FIGURE 1:**
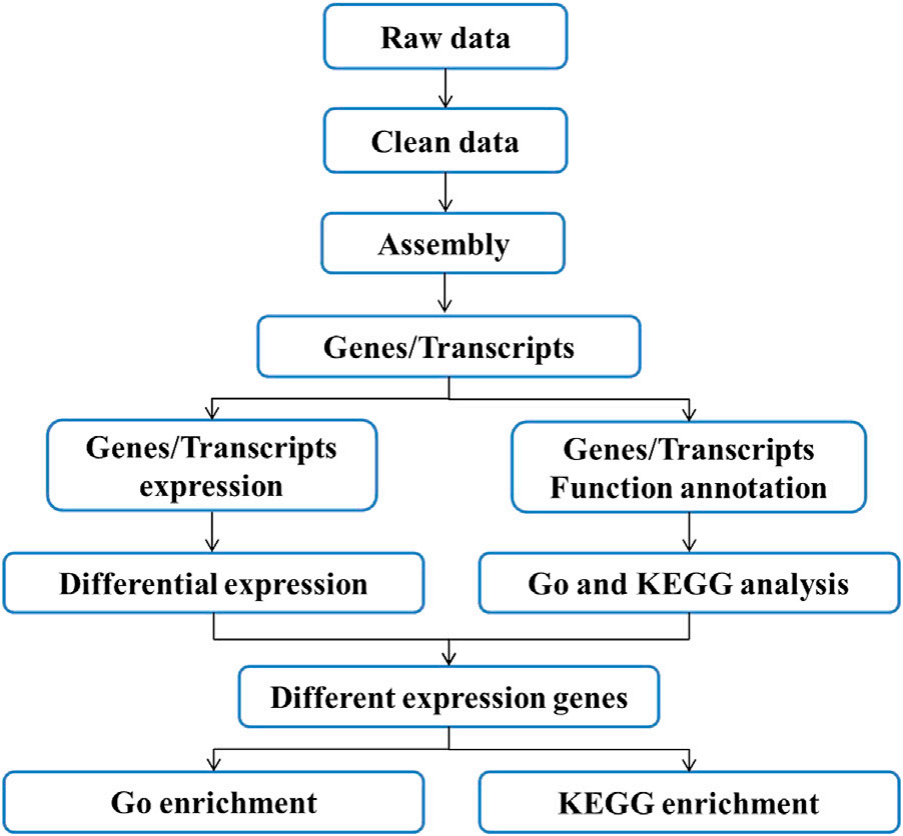
Detailed schematic diagram of the bioinformatics pipeline.

The S. *divaricata* roots of Umbelliferae plants were selected as the test materials. Samples of S. *divaricata* were collected from its cultivation base in Baicheng City, Jilin Province (E122.51, N45.37) at the end of September 2020. Single and double-headed root plants were still grown under the well-cultivated conditions. Those were dug out from the soil, all root tissues from every 3 individual plants were mixed as one biological replicate to be stored in liquid nitrogen. Three biological replicates were respectively collected from the treatment and control groups for transcriptome sequencing. Six biological replicates were respectively collected from the treatment and control groups for metabolomic sequencing. The remaining samples were dried at 45°C to a constant weight, mixed and crushed, for determinating ginsenoside content. The differences between plants with single- and double-headed roots are shown in Figure 1.

### Determination of the Chromone Content

After drying the samples, these were ground into a powder. The ground powder was filtered through a 60-mesh sieve and then weighed in three replicates, each weighing 1.0 g. Chromogen ketones were extracted ultrasonically (extraction conditions: ultrasonic frequency, 40 kHz; extraction temperature, 30°C; extraction time, 30 min; liquid ratio, 1:15), extracted three times with methanol, filtered in a funnel, and then the filtered liquids were combined. After the filtrate was evaporated in an evaporating dish and transferred to a 5 ml volumetric flask, the volume was adjusted to 5 ml and the solution was filtered using a 0.22 μm filter. High-performance liquid chromatography was performed using an Agilent 1,200 series high-performance liquid chromatography high-performance liquid chromatography system (Agilent, Palo Alto, CA, United States), equipped with an autosampler and a UV detector with a C18 column (4.6 mm × 250 mm, 5 μm; Agilent). Gradient elution was performed using solvent A (100% methanol) and solvent B (100% water) at 30°C, according to the following gradient program: 0–20 min, 30% A; 20–25 min, 45% A; 25–50 min, 60% A; 50–55 min, 90% A; and 55–60 min, 100% A. The flow rate was maintained at 1.0 ml/min, the sample injection volume was 10 μl, and UV absorption was measured at 254 nm. Quantitative analysis was performed using the one-point curve method using an external standard of authentic chromones (prim-*O*-glucosylcimifugin, 4′-*O*-β-*D*-glucosyl-5-*O*-methylvisamminol, cimifugin, and sec-*O*-glucosylhamaudol) and the following equations:

$$y=5013.2x–4988.3\;\left(r^{2}=0.9983\right)\;$$

$$y=7810.9x–5120.6\;\left(r^{2}=0.9990\right)$$

$$y=6177.9x–3435.5\;\left(r^{2}=0.9997\right)$$

$$y=2867.9x–2658.2\;\left(r^{2}=0.9992\right)$$

For prim-*O*-glucosylcimifugin, 4′-*O*-β-*D*-glucosyl-5-*O*-methylvisamminol, cimifugin, and sec-*O*-glucosylhamaudol, respectively.

### RNA Sequencing

The total RNA was extracted according to the instruction manual of TRIzol reagent (Invitrogen, Waltham, MA, United States). Bioanalyzer 2100 and RNA 1000 Nano LabChip Kit (Agilent) were used to analyze the total RNA quantity and purity. Poly(A) RNA was purified from the total RNA using magnetic beads linked to poly-T oligonucleotides. Next, at a temperature of 95°C, divalent cations were used to fragment the mRNA into small pieces. The RNA-Seq sample preparation kit (Illumina, San Diego, CA, United States) was used to reverse transcribe the cleaved RNA fragments to create the final cDNA library. The average insert size of the double-ended library was 300 ± 50 bp. Paired-end sequencing was performed by LC Sciences (Houston, TX, United States) using an IlluminaHiseq 4,000 sequencer.

### *De Novo* Assembly, Unigene Annotation, and Functional Classification

**FIGURE 2:**
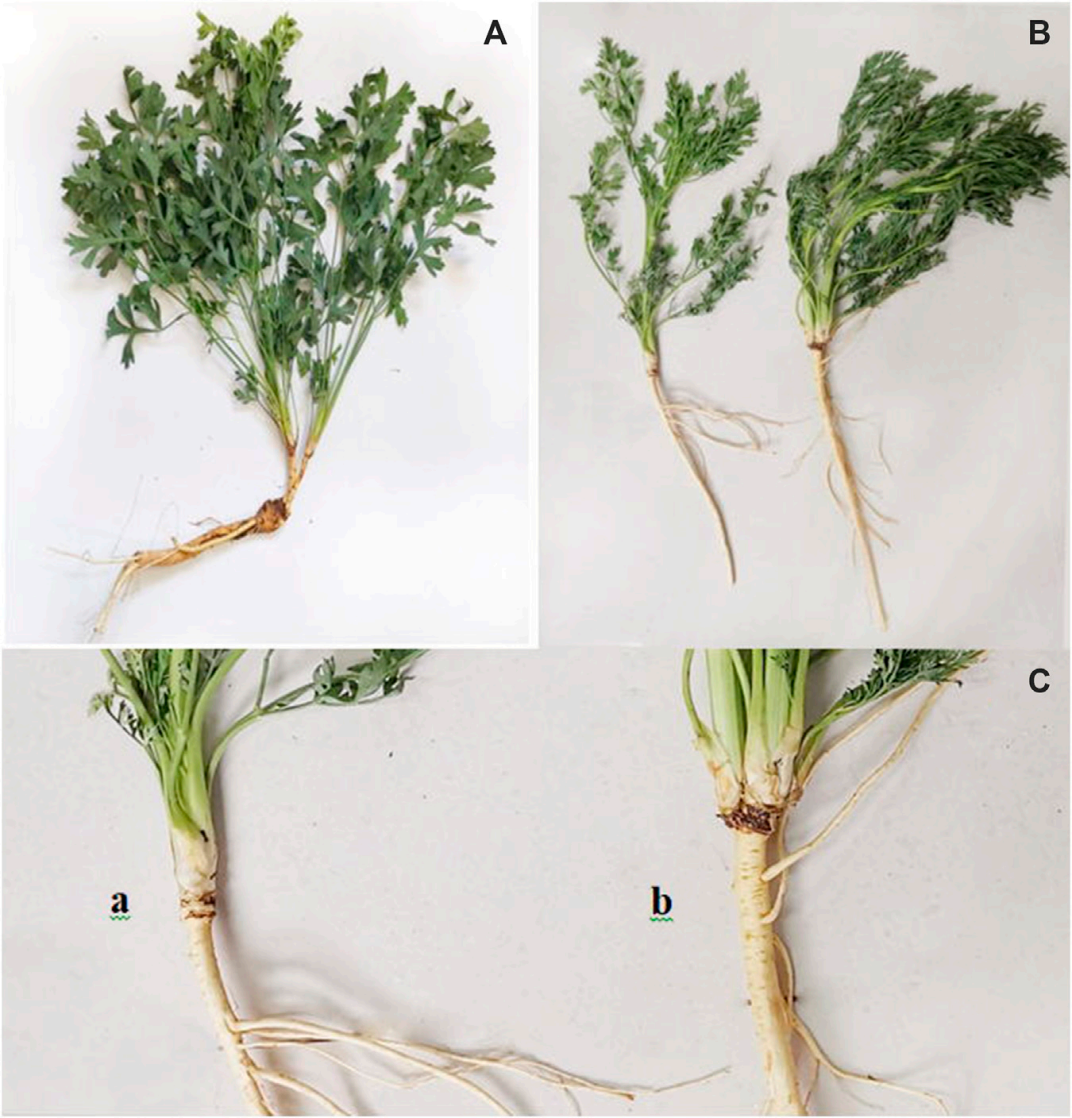
The sample of SD. Note: **(A)** is double-headed root SD; **(B)** is the whole plant of single-headed root and double-headed root; In **(C)**, a is a single-headed root, and b is a double-headed root.

The workflow of the bioinformatics analysis is shown in Figure 2. Cutadapt and Perl scripts were used to delete the reads containing adapter contamination, low-quality bases, and undetermined bases (Martin, 2011). FastQC (http://www.bioinformatics.babraham.ac.uk/projects/fastqc/) was used to verify sequence quality and calculate the Q20, Q30, and GC content of clean data. All downstream analyses were based on high-quality, clean data. The *de novo* assembly of the transcriptome data was performed using Trinity 2.4.0. The transcripts were grouped into clusters according to the shared sequence content, and the transcript with the largest expansion in each cluster was selected as the unigene (Grabherr et al., 2011). All assembled unigenes were linked to the non-redundant protein database (http://www.ncbi.nlm.nih.gov/), Gene Ontology (GO; http://www.geneontology.org), SwissProt for comparison (http://www.expasy.ch/sprot/), Kyoto Encyclopedia of Genes and Genomes (KEGG; http://www.genome.jp/kegg/), and eggNOG (http://eggnogdb.embl.de/). A DIAMOND threshold E value of <0.00001 was used (Buchfink et al., 2015).

### Identification of the DEGs

Salmon (https://combine-lab.github.io/salmon/) was used to determine the expression level of unigenes by calculating the transcripts per million (Patro et al., 2017). The “edgeR” package in R was used to select the DEGs at *p* < 0.05 when the log2 (multiple change) > 1 or log2 (multiple change) < −1 (Robinson et al., 2010).

### Metabolite Extraction and Liquid Chromatography Mass-Spectroscopy

The collected samples were thawed on ice, and 120 µl of pre-chilled 50% methanol buffer was used to extract the metabolites from 20 µl of each sample. The metabolite mixture was vortexed for 1 min, incubated at 20°C–25°C for 10 min, and stored at −20°C overnight. The mixture was centrifuged at 4,000 × *g* for 20 min, and the supernatant was transferred to a 96-well plate. The samples were stored at −80°C before the LC-MS analysis. Mixed quality control (QC) samples were prepared by combining 10 μl of each extraction mixture.

### LC-MS Analysis

All test samples were analyzed using a TripleTOF 5,600 Plus high-resolution tandem mass spectrometer (SCIEX, Warrington, United Kingdom) in the positive and negative ion modes, and an ultra-high-performance liquid chromatography system equipped with Acquity ultra-high-performance liquid T3 columns (SCIEX) for separation (100 mm × 2.1 mm, 1.8 µm; Waters, Milford, MA, United States). The mobile phase consisted of solvent A (water, 0.1% formic acid) and solvent B (acetonitrile, 0.1% formic acid). The gradient elution conditions were as follows: flow rate, 0.4 ml/min: 5% solvent B, for 0–0.5 min; 5%–100% solvent B for 0.5–7 min; 100% solvent B for 7–8 min; 100%–5% solvent B for 8–8.1 min; and 5% solvent B for 8.1–10 min. The column temperature was maintained at 35°C. The TripleTOF 5,600 Plus system was used to detect the metabolites eluted from the column. The curtain gas pressure was set to 30 psi, and the pressure of the ion source gas 1 and 2 was set to 60 psi. The temperature of the interface heater was set to 650°C. For the positive and negative ion modes, the ion spray float voltage values used were 5 and −4.5 kV, respectively. MS data were collected in IDA mode. The time of flight mass range used was 60–1,200 Da. Survey scans were collected every 150 ms, and up to 12 production scans were collected if the threshold of 100 counts exceeded the 1+ charge state. The total cycle time was set to 0.56 s. The four-time periods of each scan were summed at a pulse frequency of 11 kHz by monitoring using a 40 GHz multi-channel thermal conductivity detector with four anodes/channel detection. Dynamic exclusion was set to 4 s. During the entire collection period, every 20 samples, the system was calibrated for mass accuracy, and every ten samples, one QC sample was analyzed to evaluate the stability of the LC-MS.

### Metabolomic Data Processing

XCMS was used to preprocess the obtained LC-MS data. The original data file was converted to the mzXML format and then processed using the “XCMS,” “CAMERA,” and “metal” packages in R. Each ion was identified based on comprehensive information about the retention time and m/z. The intensity of each peak was determined, and a three-dimensional matrix containing any specified peak index (retention time-m/z pair), the sample name (observation result), and ion intensity information (variable) was generated. Then, the information was matched with internal and public databases. KEGG and the human metabolome database were used to annotate the metabolites by matching their molecular weight data (m/z) within the 10-ppm threshold. The “metal” R package was used to further process the peak intensity data. The features detected in <50% of the QC samples or 80% of the test samples were deleted, and the k-nearest neighbor algorithm was used to extrapolate the values of the missing peaks to further improve data quality. Principal component analysis was performed to use the processed data set to detect any outliers and batch effects. QC data about the injection sequence were fit to QC-based local regressions to minimize the signal intensity drift over time. In addition, the relative standard deviations of the metabolic characteristics were calculated for all QC samples, and those with standard deviations >30% were eliminated. Before performing QC-robust spline batch correction, the data were normalized using a probability quotient normalization algorithm. Student’s t-test was used to determine the significant differences, and then the Benjamini-Hochberg procedure was used to adjust for multiple tests. Furthermore, the “metal” R package was used to perform a supervised partial least squares discriminant analysis using a variable importance cutoff value of 1.0 to identify the differentially expressed metabolites (DEMs) between the two phenotypes.

### Quantitative Reverse Transcription-PCR

The instructions of the TaKaRa MiniBEST Universal RNA Extraction Kit were followed for total RNA extraction (TaKaRa, Kusatsu, Shiga, Japan), and the PrimeScriptTM RT Master Mix Kit (TaKaRa) was used for reverse transcription. RT-qPCR was performed on a 96-well plate using an Agilent Technologies Stratagene Mx3000P thermal cycler and a SYBR Green-based PCR kit. Each reaction involved 1 μl of cDNA template (1 mg/ml), 10 μl of SYBR Green Mix (TaKaRa), 1 μl of forward primer (1 mM), 1 μl of reverse primer (1 mM), and 7 μl of ddH_2_O. The thermal conditions were as follows: 95°C for 3 min, followed by 40 cycles at 95°C for 5 s, 55°C for 32 s, and 72°C for 20 s. The melting curve was obtained by gradually increasing the temperature from 55°C to 95°C at a heating rate of 0.1°C/s. RT-qPCR analysis was performed using three biological replicates. *GAPDH* (glyceraldehyde-3-phosphate dehydrogenase) was used as a housekeeping gene to estimate the relative gene expression using the 2^−ΔΔCt^ method. The primer sequences used in this study are shown in Supplementary Table S1.

### Statistical Analysis

The original data were compiled using the MS Excel 2016 software (Microsoft, Redmond, WA, United States), and SPSS 19.0 was used for data analysis (IBM, Armonk, NY, United States). GraphPad Prism 6.0 (GraphPad Software, San Diego, CA, United States) and Origin 9.0 (OriginLab, Northampton, MA, United States) were used to create the graphic illustrations.

## Results

### Chromone Content

**FIGURE 3:**
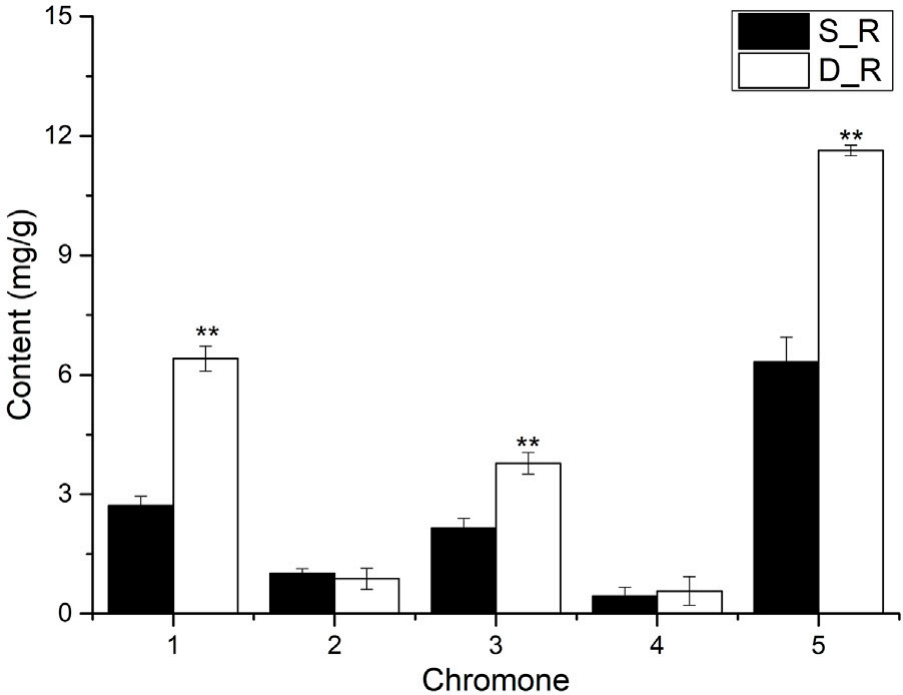
Results of chromone content in single-headed roots and double-headed roots. Note:1): prim-*O*-glucosylcimifugin; 2): 4′-*O*-β-D-glucosyl-5-*O*-methylvisamminol; 3): cimifugin; 4): sec-*O*-glucosylhamaudol; 5): Total chromone. *p < 0.05. **p < 0.01, compared with control group. Vertical bars indicate the mean value ±standard deviation from three independent experiments. The same below.

The chromone content in the single- and double-headed roots is shown in Figure 3. The prim-*O*-glucosylcimifugin, cimifugin, and total chromone contents in the double-headed roots were significantly higher (*p* < 0.01) than those in the single-headed roots, with 2.36, 1.76, and 1.84-fold differences, respectively. On the basis of the detection indicators stipulated in the 2020 edition of the Chinese Pharmacopoeia, the measurement indicators were enriched (Commission, 2020). The measurement results showed that the double-headed roots were superior to the single-headed roots in terms of the effective ingredients. Prim-*O*-glucosylcimifugin is colling and relieves pain, which are good pharmacological effects; therefore, it can be used as the first choice medicine in clinical practice.

### Transcriptomic Data Analysis

**FIGURE 4:**
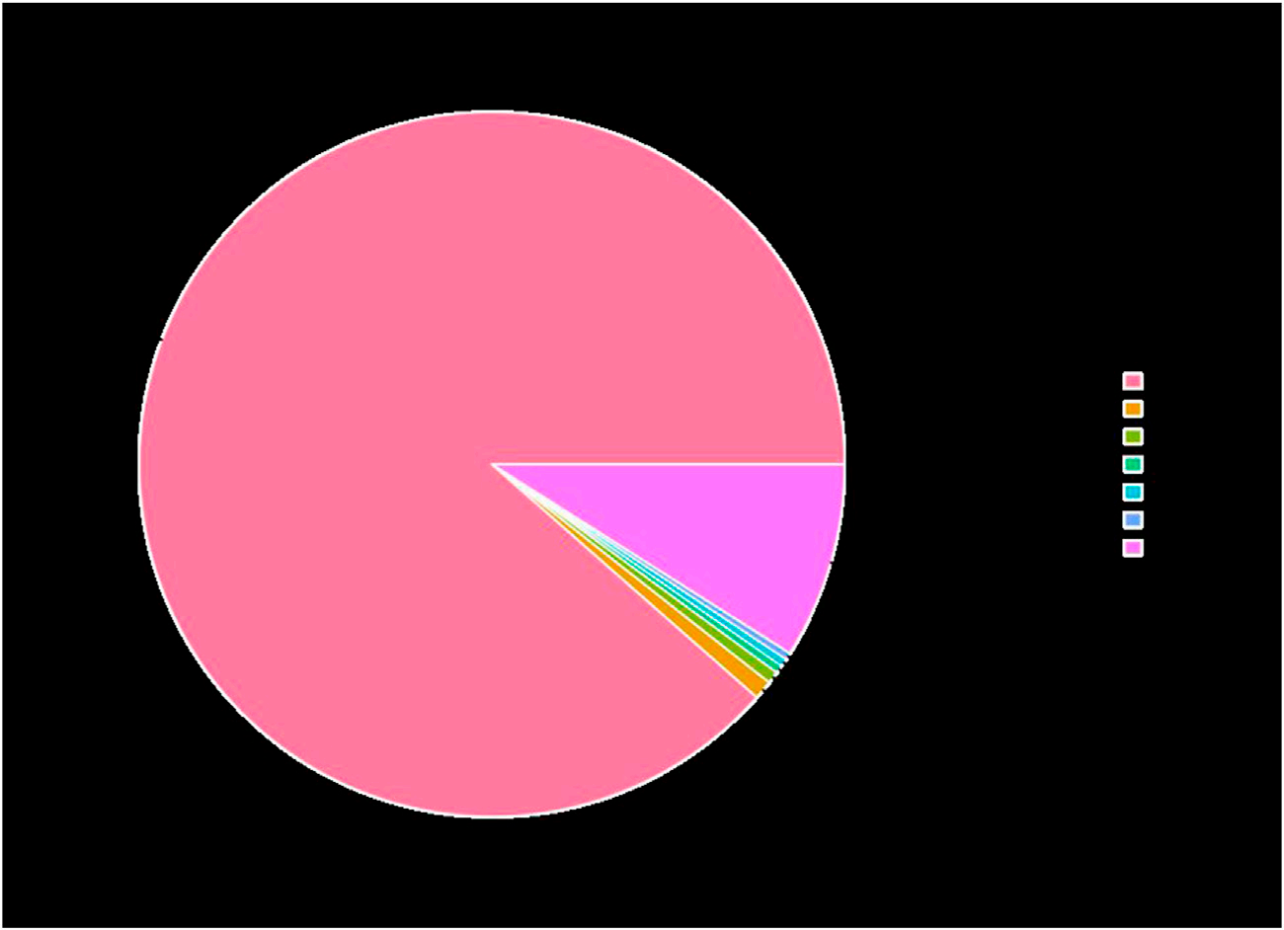
Species distribution of unigenes on NR annotation.

**FIGURE 5:**
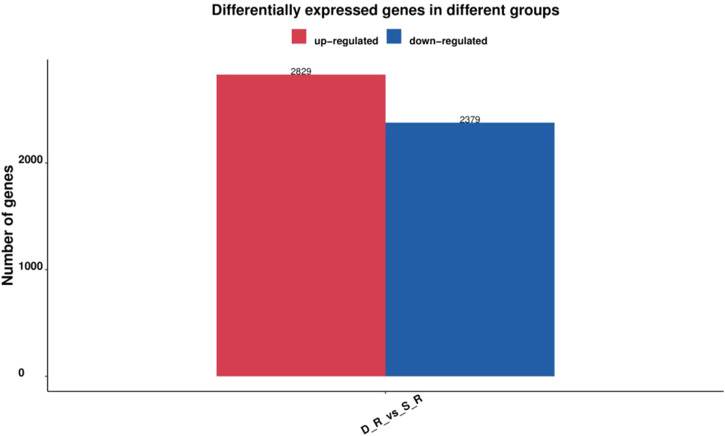
Number of DEGs in the single-head root and double-head root SD.

Six cDNA libraries from each phenotype generated 285,845,066 raw reads. After removing the adapters and filtering out the low-quality sequences, 280,693,682 clean reads were obtained from the six libraries (Table 1). After *de novo* assembly, mapping to contigs, and redundancy removal, 44,258 unigenes were obtained with an N50 length of 1,599 nucleotides (Table 2, Supplementary Figure S1). Therefore, the quality of the experimental data obtained via sequencing was high, meeting the conditions for the subsequent experimental analysis. Of the 44,258 unique sequences, 90.67% showed homology to known genes of existing models (88.51% to *Daucus carota*, 0.87% to *Quercus suber*, 0.57% to *Vitis vinifera*, 0.36% to *Actinidia chinensis*, and 0.36% to *Helianthus annuus*; Table 3 and Figure 4). Salmon was used to determine the expression levels of the unigenes. A total of 5,208 DEGs were screened in the D_R vs. S_R comparison group, of which 2,829 were upregulated and 2,379 were downregulated. The number of upregulated genes was higher than that of downregulated genes, indicating that the DEGs upregulated in the double-headed roots can be used as important candidate genes to identify the quality differences between both phenotypes (Figure 5).

**TABLE 1:** The RNA-seq data for these 6 samples.

Sample	Raw reads	Raw bases (G)	Valid eads	Valid bases (G)	Valid%	Q20%	Q30%	GC%
D_R_1	41,486,368	6.22	40,820,710	5.72	98.40	98.18	93.77	43.69
D_R_2	52,422,016	7.86	51,496,090	7.21	98.23	98.21	93.91	43.59
D_R_3	47,555,580	7.13	46,691,176	6.54	98.18	98.29	94.12	43.92
S_R_1	47,065,328	7.06	46,114,706	6.46	97.98	98.18	93.86	43.83
S_R_2	48,422,758	7.26	47,510,030	6.65	98.12	98.22	93.97	43.79
S_R_3	48,893,016	7.33	48,060,970	6.73	98.30	98.27	94.05	43.71

**TABLE 2:** Summary of assembly results of *SD.*

Index	All	GC%	Min length	Median length	Max length	Total assembled bases	N50
Transcript	103,139	40.10	201	755	15,586	108,266,339	1,599
Gene	44,258	40.18	201	610.00	15,586	43,027,640	1,617

**TABLE 3:** Summary of function annotation of *SD.*

DB	Num	Ratio (%)
All	44,258	100.00
GO	23,417	52.91
KEGG	18,487	41.77
Pfam	20,999	47.45
SwissProt	19,125	43.21
eggNOG	25,735	58.15
NR	28,726	64.91

**FIGURE 6:**
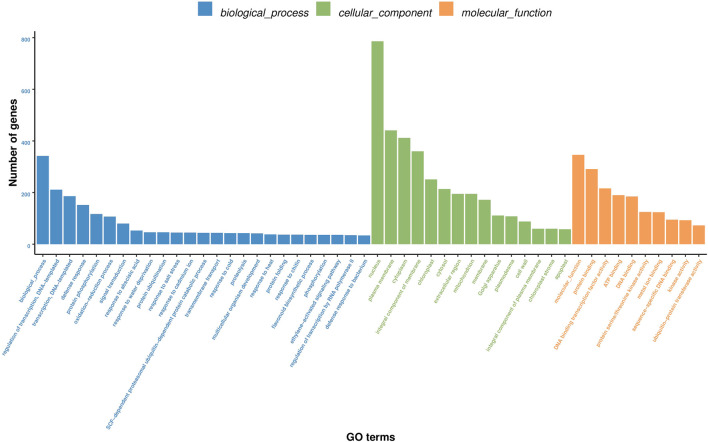
Analysis of D_R vs S_R differentially expressed gene GO enrichment.

**FIGURE 7:**
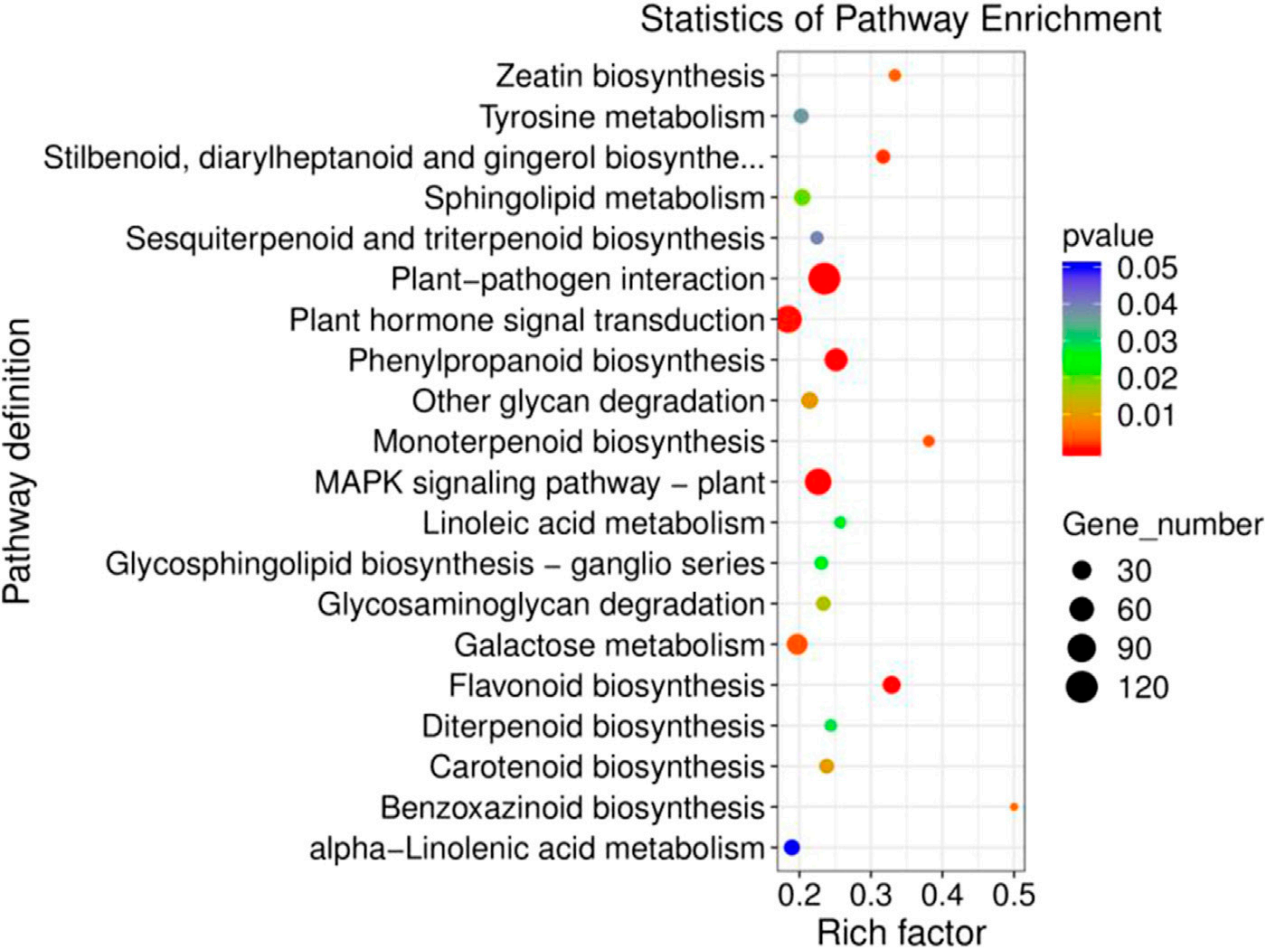
Analysis of D_R vs S_R differentially expressed gene KEGG enrichment.

**FIGURE 8:**
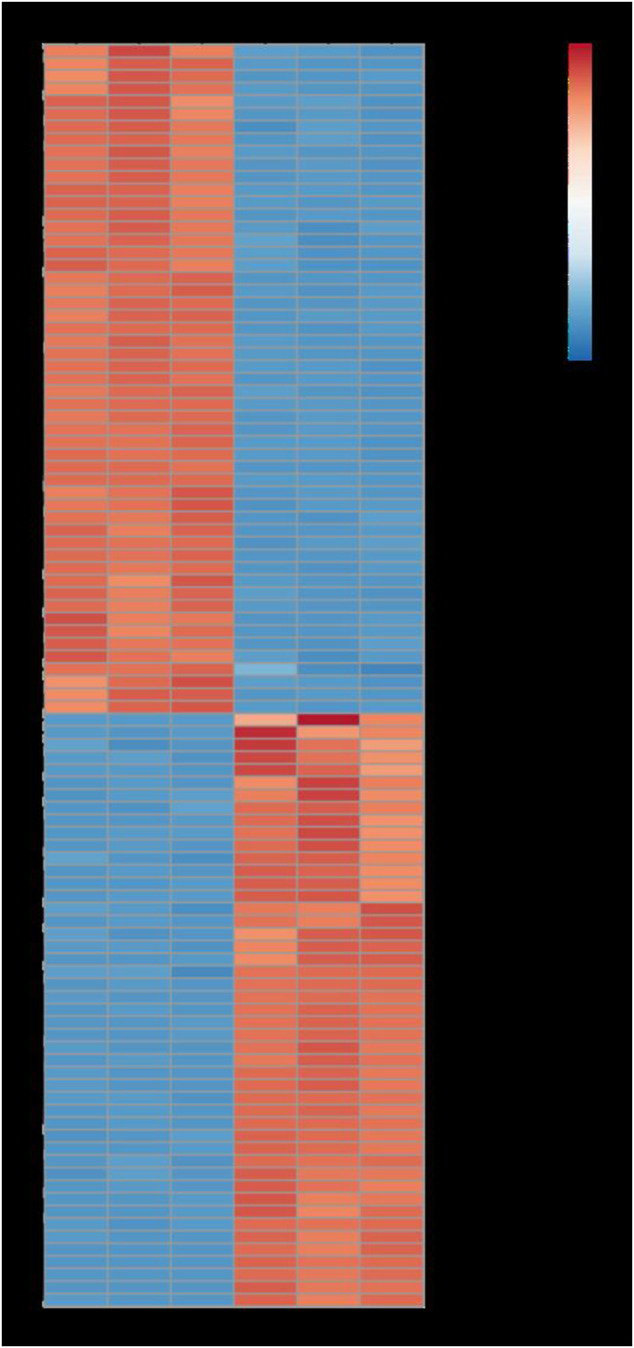
Heatmap analysis describing the differentially expressed genes. Red, up-regulated genes. Blue, down-regulated genes.

Furthermore, GO function enrichment analysis revealed that the DEGs were enriched in biological processes (i.e., biological process, regulation of transcription, DNA-templated transcription, DNA-templated defense response, and protein phosphorylation), cellular components (i.e., nucleus, plasma membrane, cytoplasm, integral components of membrane, and chloroplast), and molecular functions (i.e., molecular function, protein binding, DNA binding transcription factor activity, ATP binding, and DNA binding) (Figure 6). To further determine the internal cause of the difference in quality observed between the single- and double-headed roots (that is, the metabolic pathways associated with the quality of the medicinal material), we mapped the DEGs to the KEGG database. KEGG pathway analysis showed that the DEGs were highly associated with several pathways, including plant-pathogen interaction, plant hormone signal transduction, MAPK signaling pathway-plant, phenylpropanoid biosynthesis, and galactose metabolism (Figure 7). The DEGs were enriched in plant hormone signal transduction pathways, indicating that hormones may be essential for the quality of the medicinal materials. In addition, many genes are involved in the MAPK signaling pathway and phenylpropanoid biosynthesis-related pathways. Chromone is the most important active ingredient in *S. divaricata* and the basic framework for flavonoid synthesis. Therefore, this research focused on the synthesis pathways of plant hormones and flavonoids. Cluster analysis demonstrated that the number of upregulated genes was significantly higher than that of downregulated genes Figure 8. The test results indicated that the higher number of upregulated genes in double-headed roots may be related to the accumulation of active ingredients. This experimental result was mutually corroborated by the previous differential gene expression analysis.

**FIGURE 9:**
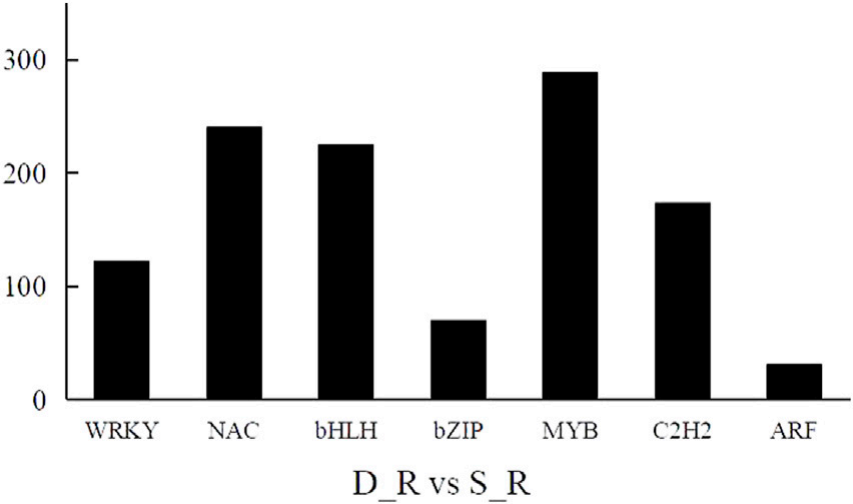
Differentially expressed transcription factor results.

A transcription factor is a protein with a special structure that regulates gene expression. Transcription factors initiate and regulate gene expression by recognizing and binding to cis-acting elements in the promoter region of genes. We also performed a statistical analysis of the differential transcription factors in the D_R vs. S_R comparison group and screened 4,166 differential transcription factors. These factors mainly belonged to the MYB (290 genes), bHLH (278 genes), NAC (241 genes), ERF (186 genes), C2H2 (174 genes), WRKY (123 genes), and bZIP (71 genes) families, as shown in Figure 9. This shows that the transcription factors of these families play an important role in the formation of double-headed root medicinal materials.

Furthermore, through the functional analysis of the differential genes and further screening, we obtained six key differential genes related to the synthesis of flavonoids and plant hormones, including five key genes in the flavonoid synthesis pathway (*4-coumarate-CoA ligase*, *chalcone synthase 1*, *vinorine synthase*, *chalcone-flavonone isomerase 1*, and *flavanone 3 beta-hydroxylase*) and one key gene in the ABA biosynthetic pathway (*zeaxanthin epoxidase*) (Table 4). Therefore, the above six genes can be used as key candidate genes to study the quality differences of double-headed root medicinal materials.

**TABLE 4:** Candidate genes differentially expressed in single-head root and double-head root SD.

Name	Gene ID	Gene expression (log2 ratio)	up/down
4CLL9	TRINITY_DN17215_c0_g5	−1.30	down
4CLL7	TRINITY_DN15920_c0_g4	−1.53	down
CHI3	TRINITY_DN13271_c0_g7	−1.42	down
4CL	TRINITY_DN12193_c0_g5	1.06	up
CHS2	TRINITY_DN12770_c0_g2	1.20	up
ZEP	TRINITY_DN6974_c0_g1	4.15	up

### Validation of the Transcriptome Data

**FIGURE 10:**
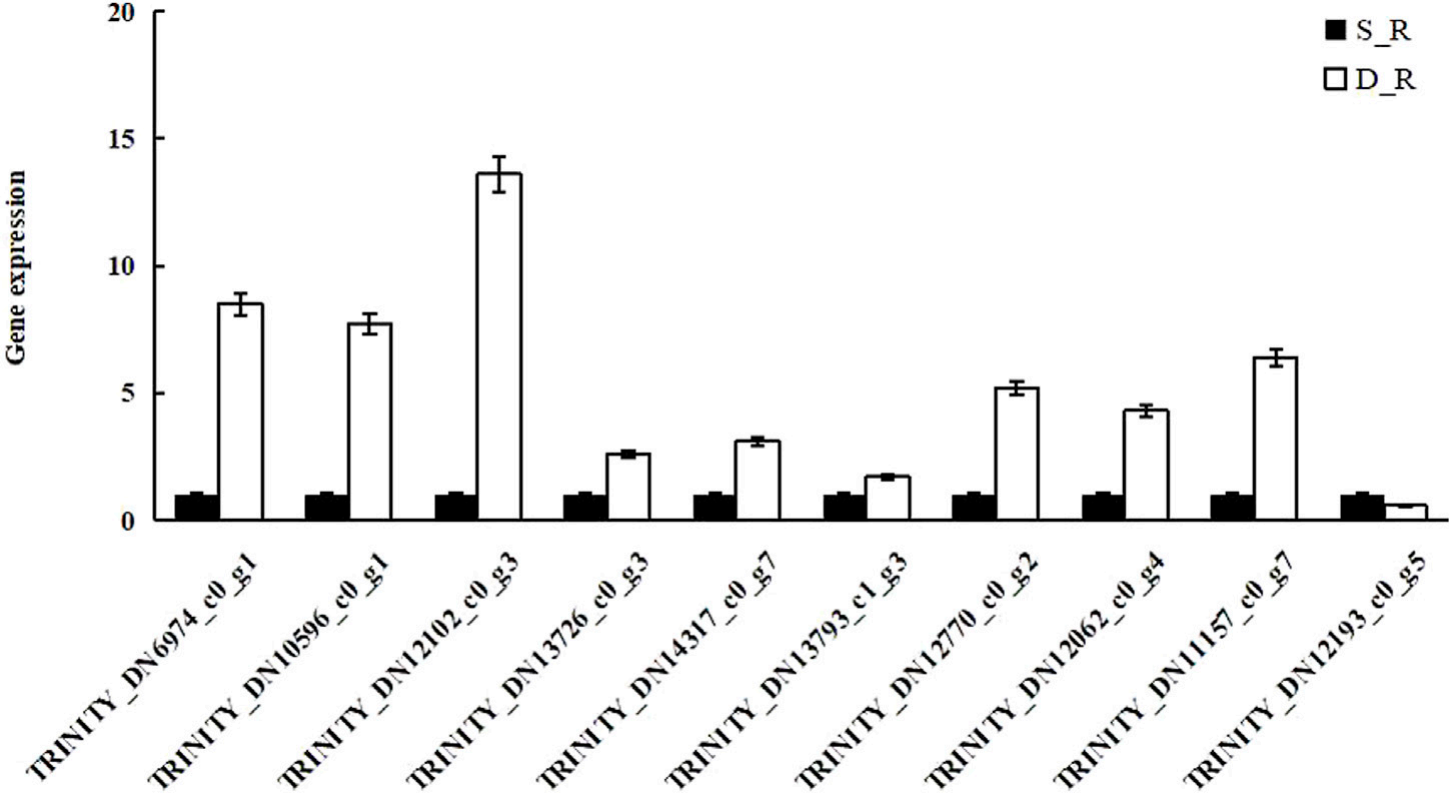
The results of transcriptome data verification.

To verify the reliability of the transcriptome data, qRT-PCR was performed for ten randomly selected DEGs. qRT-PCR was used to compare and analyze the expression of these genes between samples and the results were consistent with the transcriptome analysis results for all the targets, except for TRINITY_DN12193_c0_g5. These results are summarized in Figure 10 and support our assertion that the transcriptome sequencing data were reliable.

### Metabolomic Data and Analysis

**FIGURE 11:**
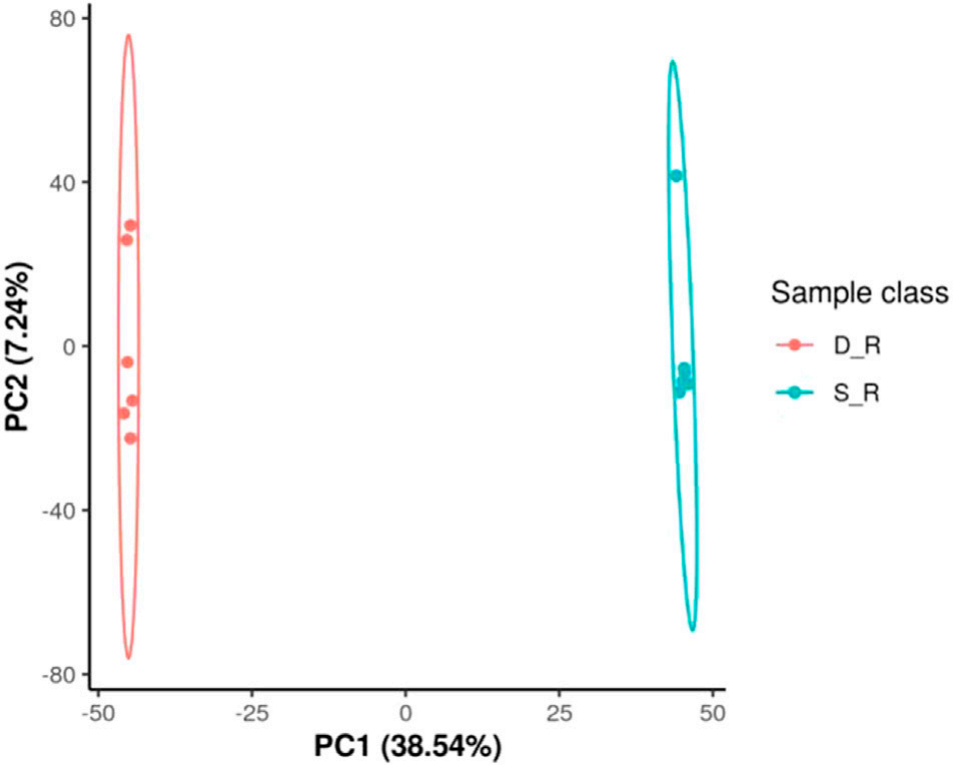
Root metabolome variation among samples as determined by principal component analysis (PCA). a Positive electrospray ionization (ESI) mode, b negative ESI mode; PC1 represents the first principal component; PC2 represents the second principal component. D_R, double-head root; S_R, single-head root; QC, quality control (a mixture of experimental samples prepared in equal amounts). The data shown are the means of six biological replicates.

**FIGURE 12:**
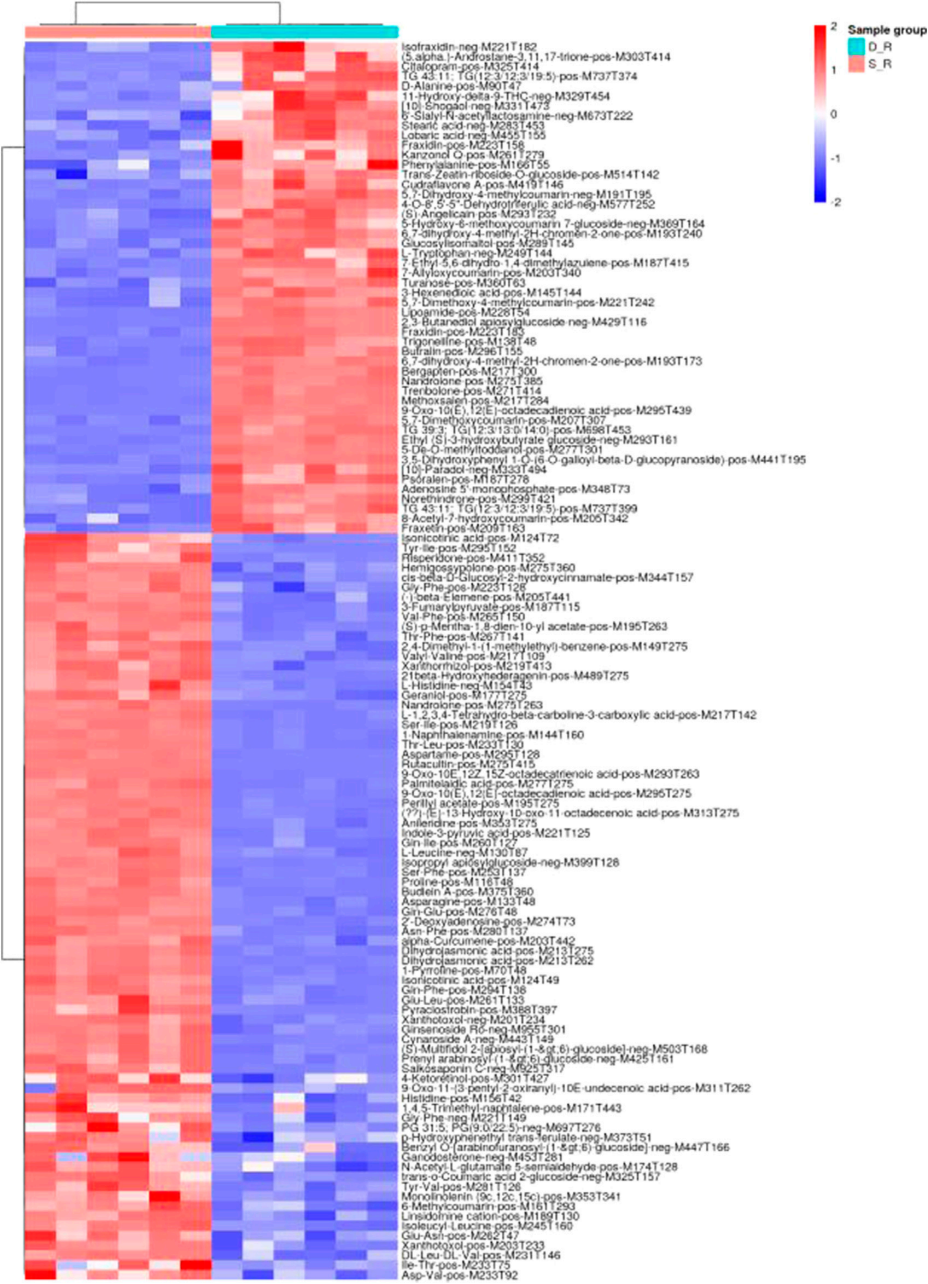
Results of hierarchical clustering analysis of altered metabolite pools in the single-headed roots and double-headed roots (*p* < 0.01). Heatmap color indicate the abundance of each metabolite in the single-headed roots and double-headed roots.

**FIGURE 13:**
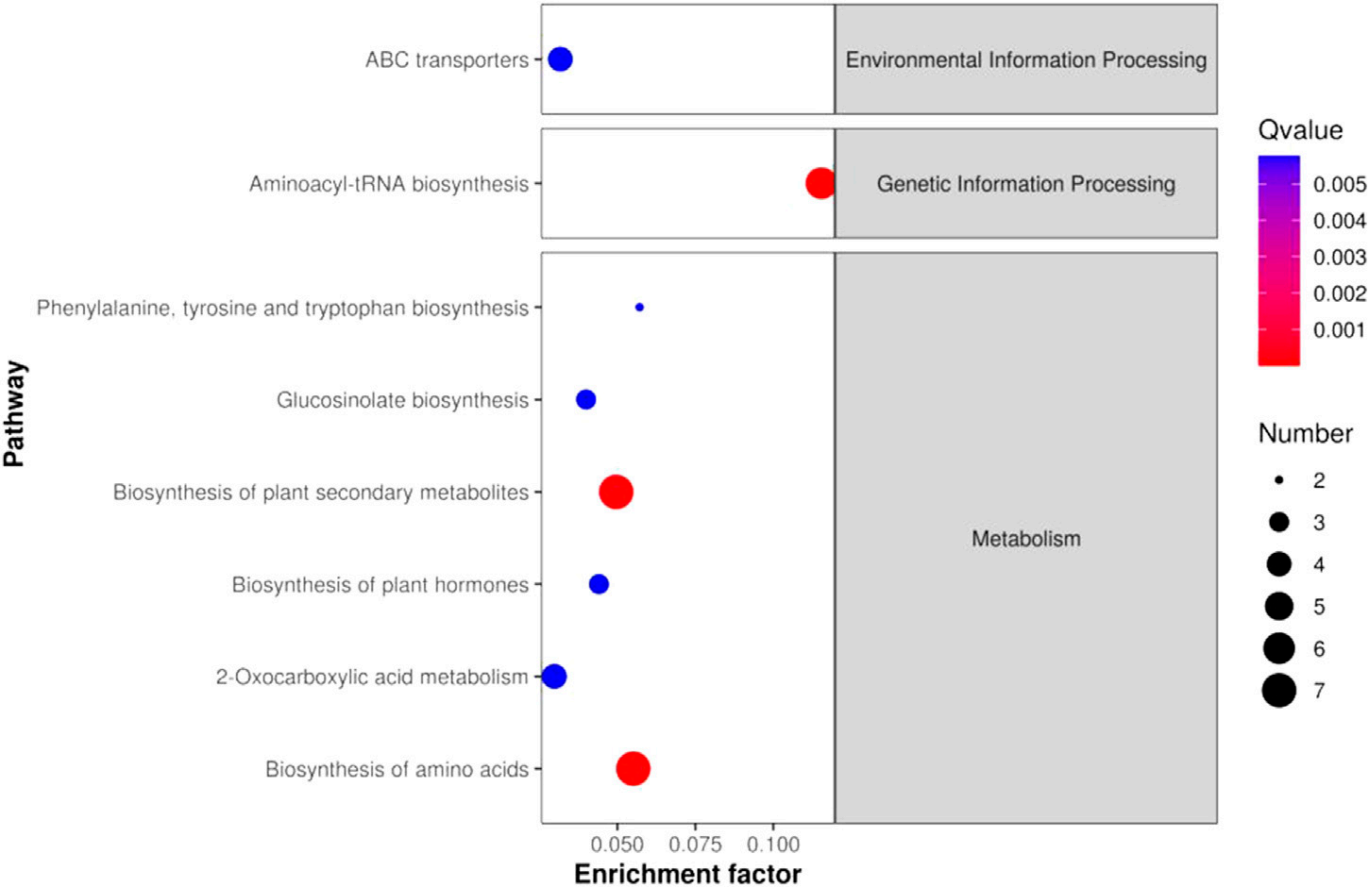
The result of differential metabolite enrichment analysis.

The analysis of the metabolomic data revealed 126 DEMs (30 organic acids and derivatives, 30 lipids and lipid-like molecules, 20 phenylpropanoids and polyketides, 11 organoheterocyclic compounds, eight organic oxygen compounds, six benzenoids, two nucleosides, nucleotides, and analogs, one alkaloid and derivatives, one hydrocarbon, one organooxygen compound, and 16 other compounds; Supplementary Table S2). According to principal component analysis for the positive electrospray ionization (ESI) mode, the first PC (PC1) and the second PC (PC2) explained 38.54 and 7.24% of the total measured metabolite variation, respectively, in the direction of the treatments. Similar results were obtained for the negative ESI mode (Figure 11). Using heat map analysis, we observed that the abundance of primary metabolites was significantly different between phenotypes (Figure 12). The major metabolites in the double-headed root phenotype were phenylpropanoids, polyketides, lipids, lipid-like molecules, organic acids, and derivatives. Moreover, the cudraflavone A and coumarin contents were 6.38 and 3.27-fold higher, respectively, in the double-headed roots than in the single-headed roots. KEGG analysis of the DEMs revealed that they were mainly enriched in phenylalanine, tyrosine, tryptophan, and glucosinolate biosynthesis, in the biosynthesis of plant secondary metabolites and plant hormones, 2-oxocarboxylic acid metabolism, and in the biosynthesis of amino acids (Figure 13).

**FIGURE 14:**
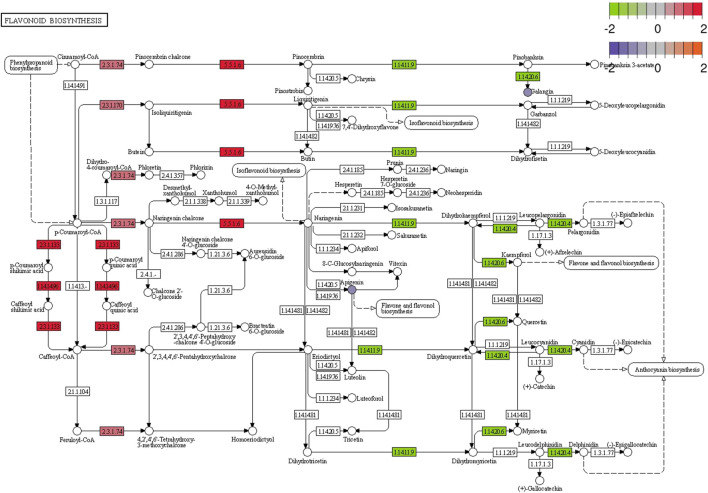
Pathway analysis of flavonoid biosynthesis pathways in single-headed roots and double-headed roots. The proposed metabolic pathways are based on the Kyoto Encyclopedia of Genes and Genomes (KEGG) database of metabolic pathways. The metabolites, written in circle, were detected in this study. Omitted metabolic processes are represented by dashed lines. The rectangle represent the genes. The red colors indicates upregulated expression and green colors indicates downregulated expression.

**FIGURE 15:**
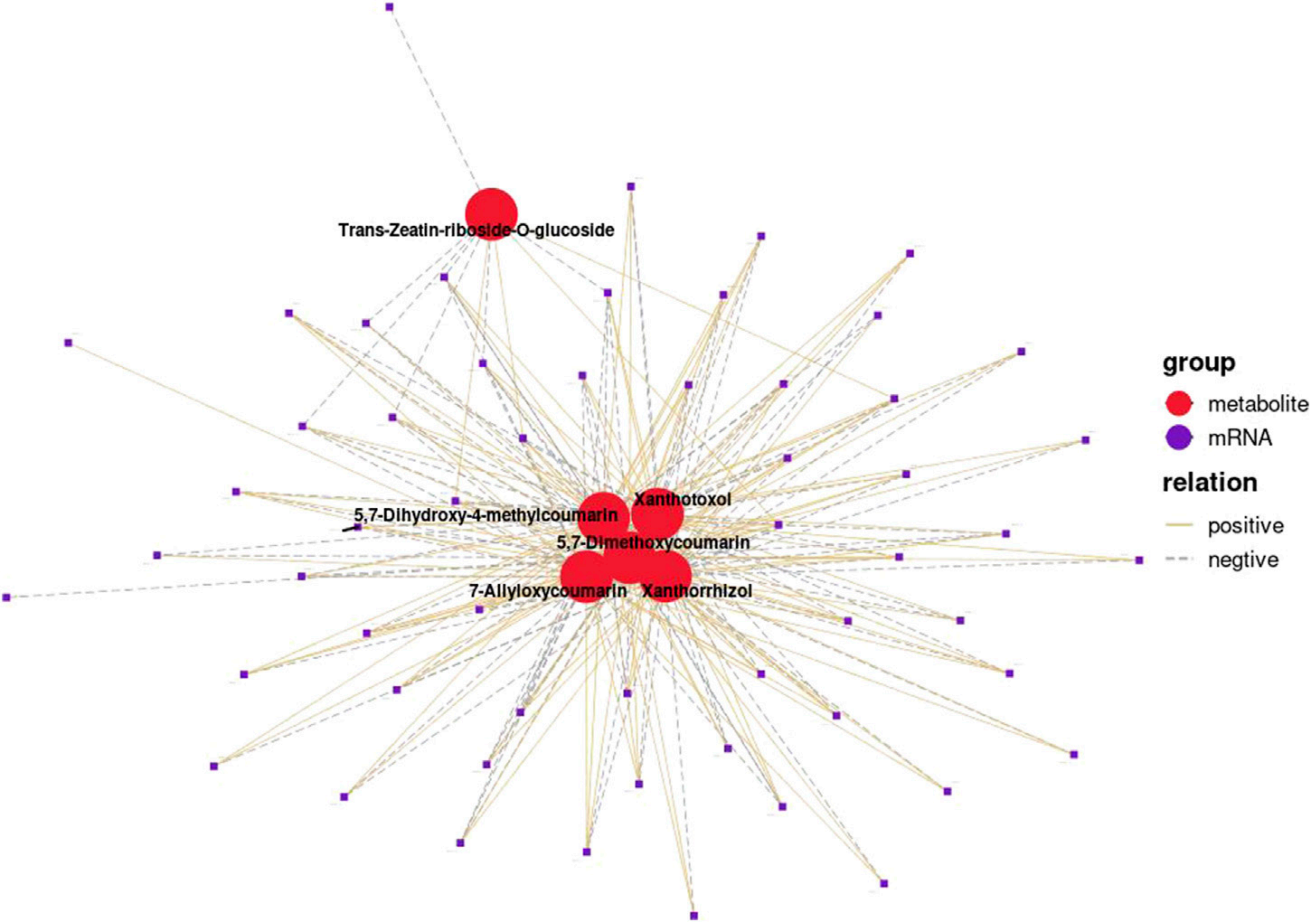
Pearson correlation analysis of differential genes and metabolites in single-head root and double-head root (*r* > 0.9, *p* < 0.01). Red circle represent metabolites, and purple circle represent genes. The same type of metabolite or gene is marked with the same color. The connection between genes and metabolites represents a correlation between the two, with yellow solid line representing a positive correlation and purple dotted line representing a negative correlation.

Correlation analysis between the DEGs and the DEMs showed that the genes associated with flavonoid synthesis, such as *flavanone isomerase* (EC 5.5.1.6), *chalcone synthase* (EC 2.3.1.74), and *vinorine synthase* (EC 2.3.1.133), were upregulated, whereas *flavanone 3 beta-hydroxylase* (EC 1.14.11.9) was downregulated in the double-headed root phenotype (Figure 14). A network diagram of the relevant genes and metabolites was constructed to analyze the correlation between the DEGs and DEMs in the two phenotypes (Figure 15). The results showed that the changes in 5,7-Dimethoxycoumarin, 5,7-Dimethoxy-4-methylcoumarin, 5,7-Dihydroxy-4-methylcoumarin, 7-Allyloxycoumarin, and *trans*-zeatin-riboside-O-glucoside were correlated with several genes.

## Discussion

Here, we assessed the morphology of *S. divaricata* with single- and double-headed roots, and tested samples for four kinds of medicinal ingredients. Simultaneously, we used transcriptomic and metabolomic analyses to identify the differences between the two phenotypes. We found that plants with double-headed roots had a superior yield potential and efficiency in accumulating organic matter. Moreover, the contents of prim-*O*-glucosylcimifugin, cimifugin, and total chromone in the double-headed root phenotype were significantly higher than those in the single-headed root phenotype. Flavonoids are a major class of secondary metabolites (Pourcel et al., 2007; Takao et al., 2021), and the chromone content is considered an important index for evaluating the medicinal value of a plant (Lv et al., 2019; Kuang et al., 2020). The active ingredients in *S. divaricata*, prim-*O*-glucosylcimifugin and 4′-O-*β*-D-glucosyl-5-*O*-methylvisamminol, can significantly reduce the body temperature of rats with yeast-induced fever and simultaneously effectively inhibit the pain caused by various stimuli in mice (Xue et al., 2000). Furthermore, research findings show that chromone has a significant antipyretic effect (Yang D.-s. et al., 2017). Double-headed roots have high contents of medicinal ingredients; thus, this phenotype might have better therapeutic properties. Therefore, it is of great significance to elucidate the internal mechanism of the quality difference between single- and double-headed root plants.

Prim-*O*-glucosylcimifugin and 4′-O-*β*-D-glucosyl-5-*O*-methylvisamminol are quality evaluation indicators, as stipulated in the 2020 edition of the Chinese Pharmacopoeia. Both belong to the chromone group of ingredients. The differential expression of the key enzyme genes (i.e., *4-coumarate-CoA ligase*, *chalcone synthase*, and *chalcone-flavonone*) in the synthesis pathway leads to differences in the contents of the medicinal components (Cheng et al., 2018). Thus, the analysis of its synthesis pathway is vital to explain the difference in the quality of medicinal materials. In the present study, transcriptome sequencing analysis results showed significant differences regarding 4-coumarate-CoA ligase, chalcone synthase, and chalcone-flavonone isomerase in the flavonoid synthesis pathway. Different copies of 4-coumarate-CoA ligase showed inconsistent expression patterns; chalcone synthase 1 was upregulated, whereas chalcone-flavonone isomerase 1 was downregulated in the double-headed root phenotype, suggesting modifications in the flavonoid biosynthesis pathway. Thus, the accumulation of active ingredients in the double-headed root phenotype might be closely related to the high expression of flavonoid synthesis genes. Phytohormones refer to trace organic matter that is synthesized to regulate various plant processes. ABA affects plant growth and development by regulating the plant and environmental signals and phytohormones, which are small molecular signal substances that transmit exogenous signals to the internal biosynthesis process of the plant (Hong and Xu, 2013). Here, the key enzyme gene of the ABA synthesis pathway, *zeaxanthin epoxidase*, was found in the double-headed root phenotype, with a 4.15-fold difference compared to the single-headed root phenotype (*p* < 0.01), leading to changes in ABA accumulation, and then transmitting the signal to flavonoids. In the synthetic pathway, the differential accumulation of the secondary metabolites of Fangfeng eventually leads to differences in the quality of medicinal materials (Zhang et al., 2010). These six genes can be used as important candidate genes to reveal the differences in the quality of the single- and double-headed root *S. divaricata* medicinal materials.

Metabolomics analysis helps identify the changes in metabolites (Hall et al., 2002), which are the final products of cell regulation processes and are regarded as responses to genetic and environmental cues (Fiehn, 2002). Therefore, metabolomic analysis enables the investigation of the relationship between biological processes and phenotypes. Furthermore, some intuitive changes can also be observed at the metabolic level (Zhang et al., 2017). Previous studies have examined the metabolic responses of different plant species, such as *Fagopyrum esculentum* (Kim et al., 2013) and *Camellia sinensis* (Yang JM. et al., 2017), and have suggested that they affect flavonoid biosynthesis. Therefore, here, we conducted a metabonomic analysis of samples based on transcriptomic sequencing. Here, we identified 126 DEMs between the two phenotypes, of which cudraflavone A and coumarin showed the biggest differences (6.38-fold and 3.27-fold, respectively). Moreover, the DEMs were mainly enriched in the biosynthesis of secondary metabolites and phytohormones. Thus, metabolomics might help elucidate the internal mechanism leading to the medicinal property differences between plants with single- and double-headed roots.

We used the transcriptome and metabolome data to identify the genes related to flavonoid biosynthesis and explain any differences between the single- and double-headed root phenotypes. Plants with double-headed roots probably have superior medicinal value than those with single-headed roots. Six DEGs were identified, including five key genes in the flavonoid synthesis pathway and one in the ABA synthesis pathway. Correlation analysis revealed that most of the DEGs were significantly related to chromone and coumarin metabolites. Moreover, *trans*-zeatin-riboside-O-glucoside, an intermediate metabolite in ABA synthesis, was also correlated with the DEGs. Thus, the accumulation of active ingredients in double-headed root plants is closely related to hormone synthesis, which is affected by flavonoid synthesis. ABA regulation in *S. divaricata* might also affect its medicinal properties.

## Conclusion

Our study showed significant differences in the quality of medicinal materials between single- and double-headed roots. The prim-*O*-glucosylcimifugin, cimifugin, and total chromone contents in double-headed roots were significantly higher than those in the single-headed roots. Using metabolome and transcriptome analyses, we identified six DEGs and 126 DEMs between the single- and double-headed root phenotypes, indicating that flavonoids and ABA synthesis pathways may play an important role in the synthesis of the active ingredients of *S. divaricata*. Furthermore, the six genes can be used as important candidate genes for analyzing the quality differences in wind-breaking medicinal materials. In general, our research provides important information on the quality differences of the *S. divaricata* medicinal materials. The double-headed root phenotype can be used as a candidate phenotype for clinical drugs, providing theoretical support for selecting and breeding *S. divaricata* medicinal materials.

## Data Availability

The original contributions presented in the study are included in the article/Supplementary Material, further inquiries can be directed to the corresponding authors.

## Supplementary Material

Supplementary Figure 1 The length distribution and GC content.

## Abbreviations

ABA, Abscisic acid; DEGs, Differentially expressed genes; DEMs, Differentially expressed metabolites*; GAPDH*, Glyceraldehyde-3-phosphate dehydrogenase; GO, Gene Ontology; KEGG, Kyoto Encyclopedia of Genes and Genomes; LC-MS, Liquid Chromatography Mass-Spectroscopy; QC, Quality control.
