# Supplementary figures and images for: Transcriptomic and Metabolomic Differences Between Two Saposhnikovia divaricata (Turcz.) Schischk Phenotypes With Single- and Double-Headed Roots
Source: Front Bioeng Biotechnol. 2021 Oct 28;9:764093. doi: 10.3389/fbioe.2021.764093 (PMC8581353; doi:10.3389/fbioe.2021.764093)

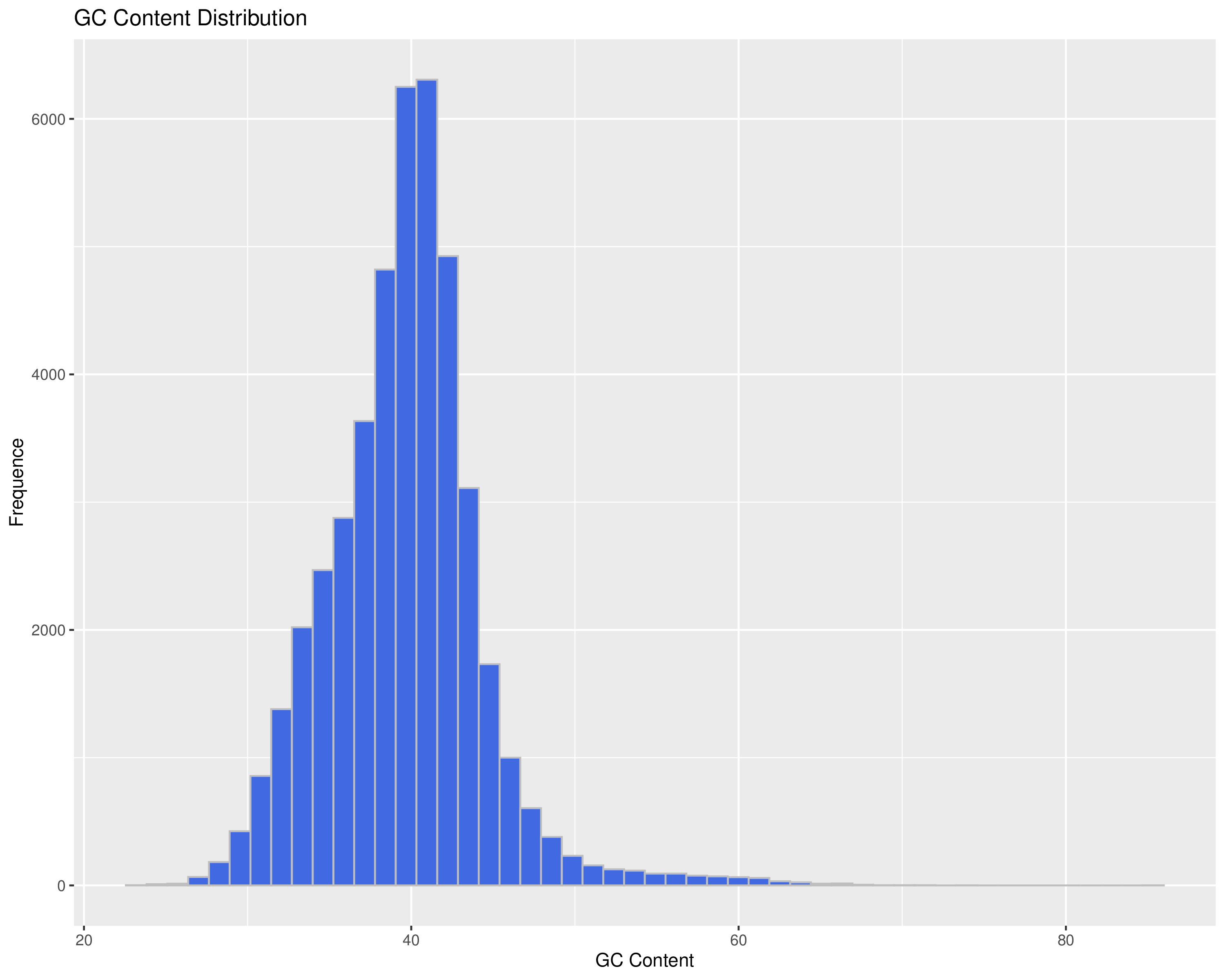

Supplement: Supplementary file 2 [file Image1.jpg]
